# The Dynamics of T-Cell Receptor Repertoire Diversity Following Thymus Transplantation for DiGeorge Anomaly

**DOI:** 10.1371/journal.pcbi.1000396

**Published:** 2009-06-12

**Authors:** Stanca M. Ciupe, Blythe H. Devlin, M. Louise Markert, Thomas B. Kepler

**Affiliations:** 1 Center for Computational Immunology, Department of Biostatistics and Bioinformatics, Duke University Medical Center, Durham, North Carolina, United States of America; 2 Department of Pediatrics, Duke University Medical Center, Durham, North Carolina, United States of America; 3 Department of Immunology, Duke University Medical Center, Durham, North Carolina, United States of America; Emory University, United States of America

## Abstract

T cell populations are regulated both by signals specific to the T-cell receptor (TCR) and by signals and resources, such as cytokines and space, that act independently of TCR specificity. Although it has been demonstrated that disruption of either of these pathways has a profound effect on T-cell development, we do not yet have an understanding of the dynamical interactions of these pathways in their joint shaping of the T cell repertoire. Complete DiGeorge Anomaly is a developmental abnormality that results in the failure of the thymus to develop, absence of T cells, and profound immune deficiency. After receiving thymic tissue grafts, patients suffering from DiGeorge anomaly develop T cells derived from their own precursors but matured in the donor tissue. We followed three DiGeorge patients after thymus transplantation to utilize the remarkable opportunity these subjects provide to elucidate human T-cell developmental regulation. Our goal is the determination of the respective roles of TCR-specific vs. TCR-nonspecific regulatory signals in the growth of these emerging T-cell populations. During the course of the study, we measured peripheral blood T-cell concentrations, TCR*β* V gene-segment usage and CDR3-length spectratypes over two years or more for each of the subjects. We find, through statistical analysis based on a novel stochastic population-dynamic T-cell model, that the carrying capacity corresponding to TCR-specific resources is approximately 1000-fold larger than that of TCR-nonspecific resources, implying that the size of the peripheral T-cell pool at steady state is determined almost entirely by TCR-nonspecific mechanisms. Nevertheless, the diversity of the TCR repertoire depends crucially on TCR-specific regulation. The estimated strength of this TCR-specific regulation is sufficient to ensure rapid establishment of TCR repertoire diversity in the early phase of T cell population growth, and to maintain TCR repertoire diversity in the face of substantial clonal expansion-induced perturbation from the steady state.

## Author Summary

Protective adaptive immunity depends crucially on the enormous diversity of the T-cell receptor repertoire, the antigen receptors expressed collectively on T-cell populations. T cells develop from T-cell precursors that originate in the bone marrow and migrate to the thymus, where their T cell receptors are constructed stochastically, and tested for autoreactivity against a host of self antigens. Complete DiGeorge anomaly is a rare congenital disease in which the thymus fails to develop, blocking all T cell development and causing profound immunodeficiency. Thymus transplantation, performed in the first two post-natal years, allows the patient's own T cell precursors to develop in the engrafted thymus tissue into normal, functioning T cells. In addition to saving patients' lives, this procedure provides an extraordinary opportunity to study the *de novo* development of human T cell populations. We have developed a mathematical model to aid in the statistical analysis of the precious data from these patients. In addition to helping elucidate the means by which the size and diversity of T cell populations are jointly regulated, the insights gained from this study hold promise for the development of therapies to promote immune recovery after transplantation.

## Introduction

An essential characteristic of T lymphocytes is their ability, as a population, to recognize an enormous number of peptide antigens. This capability is essential to the function of the adaptive immune system and is attributable to the diversity of the T-cell receptors (TCR) they express. This diversity in turn comes about through the stochastic assembly of TCR from genomic “libraries” of gene segments for the TCR alpha and beta chains, the two polypeptides that together make up the most common form of the TCR [1]–[3]. This rearrangement process takes place in the thymus and is the ultimate source of TCR repertoire diversity, though many other forces shape the raw materials thus provided [4]. It is with these other forces that we are concerned in this study.

The peripheral T-cell population is maintained at a constant size in spite of substantial, continual turnover, ongoing thymic production, and clonal expansion in response to immunological challenges [5]–[8]. Although a detailed understanding of T-cell homeostasis is not yet complete, it is clear that the maintenance of T cell population size results from the interplay of several mechanisms acting both to enhance population growth at low population density and to limit population growth at high population density [9],[10]. In a healthy individual, and in the absence of overt immune activation, the rate of T cell division in the periphery is small and is regulated through competition for resources including growth signals. In a T cell-deficient host, however, so-called lymphopenia-induced proliferation rapidly restores the system to steady-state numbers [8].

Diverse lines of investigation imply that signals delivered, and resources provided, through both TCR-specific and TCR-nonspecific channels are essential for the establishment and maintenance of the size and diversity of T cell populations [11],[12]. Experiments performed thus far have been performed in non-human animals and represent limiting cases in which one or more of these signals or resources is entirely eliminated. We are interested in understanding the phenomena in humans and in a more natural setting, in which regulation via both TCR-specific and TCR-non-specific mechanisms are intact and in their interplay jointly determine the size of the T cell population and the diversity of the TCR repertoire.

Our intent in this paper is to elucidate the mechanisms that lead to expansion and diversification of the TCR repertoire in a recovering T-cell-deficient host: in our case, a complete DiGeorge subject that has received an unrelated, unmatched thymus transplant. After transplantation, host T-cell precursors migrate from the bone marrow to the donor thymus where they develop via thymopoiesis into host T cells that then emigrate into the peripheral blood. Here, T cells have the capacity to undergo spontaneous clonal expansion, thus leading to restoration of the peripheral T-cell pool. The expansion continues until a steady state is reached. Throughout this process, selective pressure for survival emerges among and within the clones through competition for stimulatory signals.

### T Cell Homeostasis

The steady state T-cell population size arises in the balance among several phenomena, including the rapid and extensive expansion of rare clones through activation-induced peripheral division and their subsequent contraction (which constitute, in part, the T-cell immune response) and the relatively slow turnover of a diverse pool of naive cells through continuous thymic emigration and cell death. The specific memory T cells that arise as the result of clonal expansion appear to be regulated largely independently of the naive cells [11]. T cells arising through lymphopenia-induced proliferation acquire markers that ordinarily indicate a memory phenotype and may be regulated as memory cells, though this hypothesis has not been definitively tested.

Both the size and diversity of peripheral T cell populations are controlled through competition for limiting resources. (In the interest of simplicity, we will use the term “resources” familiar from ecological population studies to refer to all of the factors that mediate growth regulation. We do not intend by this to exclude factors that are more accurately referred to as “signals”) [5],[13], and may also be controlled directly by the activities of regulatory T cells [14]. It has been shown, for example, that normal T-cell population growth is dependent on stimulation by self-peptide major histocompatibility complex (spMHC) complexes through the TCR [15]–[18], requiring a TCR that is specific for the spMHC complex. But T-cell population growth also depends on cytokines such as IL7 and IL15 that act independently of TCR specificity [5], [19]–[21]. Furthermore, growth and survival in all cells require adequate space and nutrients, the utilization of which is independent of TCR specificity [22],[23].

Our current understanding of lymphopenia-induced proliferation is due to studies in mice demonstrating that T cells divide rapidly after transfer into T cell-deficient (usually due to RAG or CD3 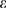 deficiency) or irradiated mice, but not after transfer to normal mice [15],[16]. Moreover, overall T cell numbers in T cell-deficient animals after transfer and clonal expansion are similar to T cell numbers in normal animals, suggesting control mechanisms acting on total T cell numbers, rather than in a clone-specific manner.

The importance of TCR specific signals has been studied at length, showing that competition within T cell clones is important in maintaining TCR repertoire diversity. It has been shown, for example, that in a T-cell-deficient host, a T cell must interact with antigen-presenting cells bearing the MHC allele responsible for that cell's thymic selection in order to proliferate [24]. In a T-cell sufficient host, such TCR-spMHC interaction is necessary for T cell survival [24],[25]. Furthermore, naive polyclonal T cells divide when transferred to TCR-transgenic hosts, as do monoclonal T cells transferred to TCR-transgenic hosts of differing clonotype. T cells do not divide, however, in hosts of identical clonotype [26]. Mice lacking MHC class II expression do not repopulate the periphery with CD4 T cells at all, suggesting that peripheral MHC class II expression is needed for the survival of CD4 T cells [25]. In the present context it is important to note that MHC class II matching in thymic grafts for complete DiGeorge subjects is not necessary for the development of CD4 T cells [27].

TCR-nonspecific signals include cytokines such as the cytokine interleukin-7 (IL7), which is necessary for the survival of nave T cells [28]–[32]. Lymphopenia-induced proliferation of memory cells requires IL7 or IL15 [13],[33]. T cells that have lost the ability to respond to IL7 after leaving the thymus are no longer able to proliferate, produce cytokines, or acquire memory cell phenotype [30]. In mice in which IL7 signaling has been completely abrogated, the few mature T cells found in the peripheral blood behave abnormally [30],[34],[35]. Experiments in IL7-receptor 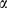 (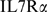)-deficient mice (IL7R^−/−^) have shown a reduction in T-cell capacity to proliferate upon stimulation, leading to a six- to seven-fold reduction in the frequency of clonogenic T cells compared with T cells from IL7R-sufficient mice (IL7R^+/+^), as well as a 50% reduction in the average clone size of single IL7R^−/−^ T cells compared with the IL7R^+/+^ T cells [34]. In another study, mice lacking the interleukin 2 (IL2) receptor 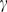 chain (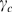) and/or the Jak family tyrosine kinase (Jak-3) had severe combined immune defects with lack of T lymphocyte maturation and function. This phenomenon is presumably attributable to the fact that 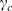 is part of the receptor for IL7 and IL15 [36]; its loss leads to the abrogation of both of these cytokine signaling pathways [30],[35], and others as well. Moreover, in humans, in the absence of of 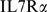 or of the Jak-3 family, the periphery lacks T cells completely [37],[38].

To assess the contributions of thymic emigration rate on the steady-state T-cell population size, Berzins et al [39] engrafted variable numbers of thymuses into mice and observed that the size of the naive T-cell population increased in proportion to the number of thymic grafts, while the size of the memory population remained unchanged. It may be of importance to note that the thymus grafts themselves produced IL7. In humans, transplantation of thymic tissue at varying doses into complete DiGeorge anomaly subjects showed no significant effect on the nave CD4 or CD8 T cell numbers [27].

### Complete DiGeorge Anomaly

Complete DiGeorge Anomaly (cDGA) is a congenital condition, the hallmark of which is profound immunodeficiency arising from abnormal development of the third and fourth pharyngeal pouches resulting in thymic aplasia (complete absence of the thymus). This developmental irregularity may also cause various other anatomical abnormalities including heart defects, hypoparathyroidism, and craniofacial malformations [40]–[42]. In the absence of a thymus, cDGA subjects lack thymic-derived T cells and are consequently profoundly immunocompromised. Postnatal thymus transplantation can lead to a restoration of T-cell function and the development of a peripheral T-cell population with an apparently normal T-cell receptor repertoire [43]–[45].

Thymus transplantation is emerging as a valuable treatment for athymia generally. There is therefore substantial medical interest in elucidating the immunological recovery of thymus transplant patients quite apart from the basic immunology these patients may reveal.

**Figure 1:**
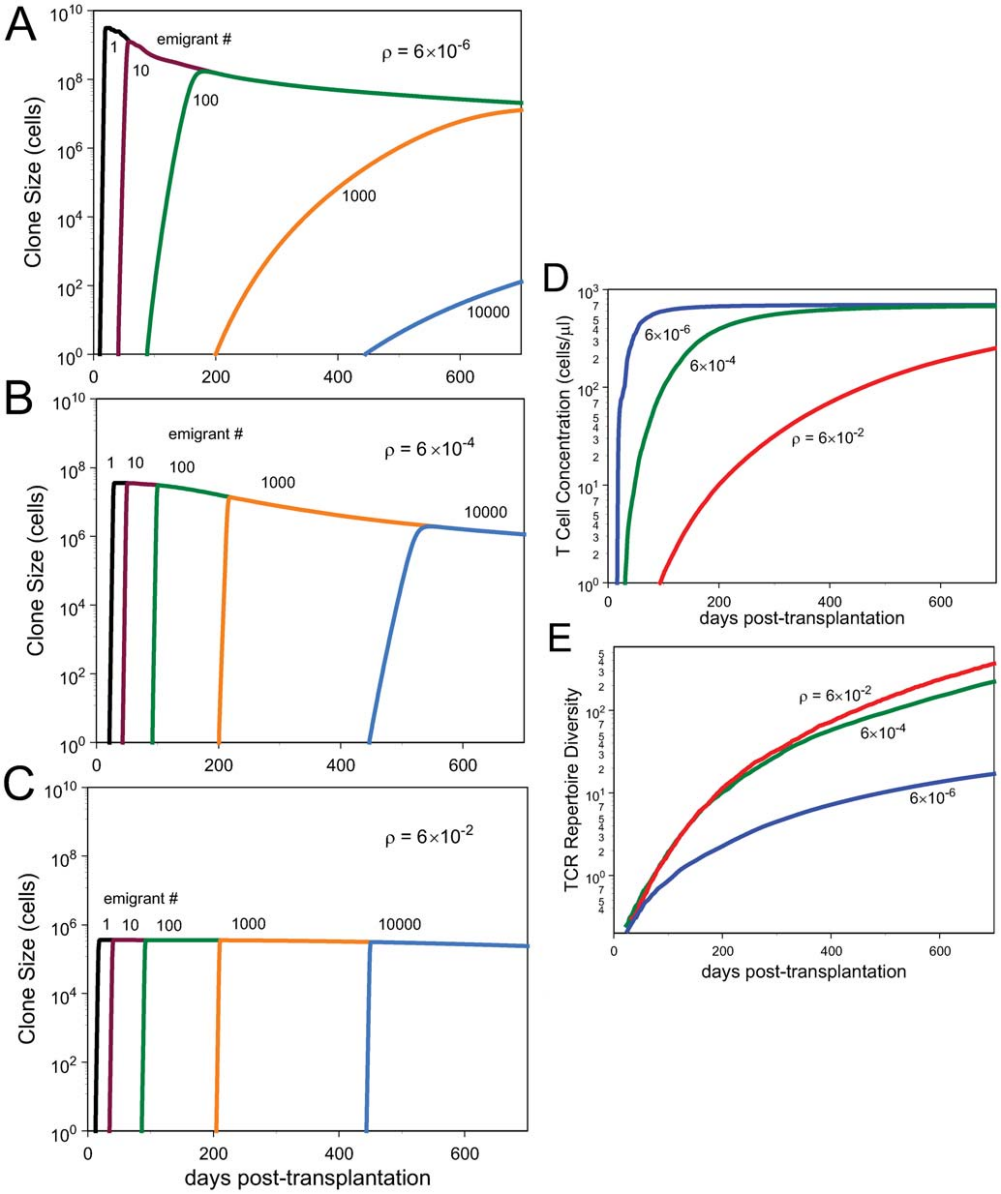
Clone sizes, T cell concentrations and TCR repertoire diversity. Clone sizes over time as computed under the model of Eqs.(1,4) (A–C) and the corresponding T cell concentrations (D) and TCR repertoire diversity (E) as a function of time past transplantation. computed under the model of Eqs.(1,4) as a function of time past transplantation. Parameter 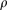 (see *Model* section) is varied as indicated while all other parameters are held constant. The higher 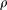, the more limiting are TCR-specific resources. “Emigrant number” refers to the order in which clones leave the thymus and enter the periphery; emigrant number 1 is the first clone to arise, etc.

To illustrate the connection between these alternative homeostatic mechanisms and the observations one might make on the DiGeorge subjects, consider the following two scenarios as a thought experiment. First, suppose that TCR-specific resources such as spMHC are not limiting, either because they are produced in great excess or because they are rendered unnecessary. Under these conditions, homeostasis will be due exclusively to competition for TCR-nonspecific resources. The first T cells leaving the thymus will expand rapidly, consuming the TCR-nonspecific resources required for growth of their own growth and for the growth of all other clones. Subsequent T cells leaving the thymus will encounter a more impoverished environment and will grow more slowly, leading to early dominance of one or a small number of early clones and therefore a limited TCR repertoire (Figure 1). Conversely, if TCR-nonspecific resources such as cytokines were not limiting, and that homeostasis therefore depended solely on competition for TCR-specific signaling, the only cellular competition would be among T cells of the same clonotype. In this case, each clone will grow to roughly the same self-limiting size regardless of when its founder emigrates from the thymus. In this case, TCR repertoire diversity will grow at the greatest possible rate, all other things being equal (Figure 1).

Reality will lie somewhere between these two limiting cases: TCR repertoire diversity will be shaped by both TCR-specific and TCR-nonspecific resources. Our goal is to explore, quantitatively, how these two sets of signals interact dynamically to shape the mature functional T cell population.

The quantitative contributions of different signals in the regulation of T-cell number and the diversity of the TCR repertoire are very difficult to determine experimentally in the context of an intact system. To examine the interplay among the mechanisms responsible for the heterogeneity of the T-cell repertoire, we have developed a mathematical model for the effective interactions among T-cell clones. We model the temporal evolution of T-cell clones and their dynamics under the combined effect of TCR-specific and TCR-nonspecific signals. In particular, we consider competition within clones for spMHC complexes presented by antigen-presenting cells as well as competition among cells in all clones for cytokines, space and other TCR-non-specific resources.

Such mathematical models have been used extensively to study the dynamical interactions between different branches of the immune system [46] and their effect on T cell [47]–[51] and B cell population development [52]. In this paper, we develop a model to examine competitive interactions among homo- and heterospecific T cells under limiting spMHC and TCR-nonspecific resources. This model then provides the basis for the analysis of clinical data from complete DiGeorge subjects following thymic transplantation to estimate the extent to which regulatory mechanisms working through these distinct pathways contribute to shaping the T cell population. The distinguishing feature of our model is its explicit representation of individual T cell clones, and the direct focus on the interplay between T-cell population size and TCR repertoire diversity this strategy allows. All model parameters are fit to data gathered within the present study; there are no parameters estimated from external sources.

## Model

We use a population-dynamic model for the analysis of patient data. The state of the model at any time is given by the variables *x_i_*, 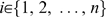 specifying the respective sizes of the 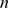 distinct effective T-cell clones. We refer to these cell subpopulations as “effective” clones because they are defined functionally, rather than genetically, in terms of the TCR-specific resources required for their survival. All T cells compete for TCR-nonspecific resources, but only cells within the same effective clone compete with each for spMHC.

The maximum growth rate for model cells is denoted *γ*. We model intraclonal competition for specific spMHC within the 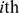 clone as cell loss at a per-capita rate 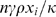 and competition for non-specific resources as cell loss at per-capita rate *γ*(1−*ρ*)*T*/*κ*. *κ* is the carrying capacity (the maximum population size sustainable in the absence of immigration) and 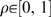 is a parameter that specifies the proportion of growth limitation attributable to TCR-specific regulation; 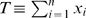 is the total size of the T cell population. We assume that all clones are equivalent–that the system dynamics are invariant under exchange of any pair of clones–and write
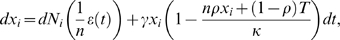
(1)where 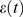 is the time-dependent total thymic emigration rate and 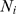 is an inhomogeneous Poisson process whose argument is the instantaneous intensity. 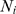 counts the number of thymic emigration events involving cells of the *i*th effective clone. In other words, the emigration of cells from the thymus is modeled as a stochastic point-process, with the times between emigration events for specificity 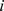 distributed as exponential random variables with mean 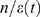. For 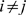, the processes 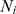 and 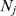 are independent.

Eq.(1) provides the basis for the numerical data fitting we perform, but analysis of the model itself is facilitated by replacing the the Poisson process in Eq.(1) by its time-dependent mean value, ie, neglecting the fluctuations due to the discreteness of the emigration process. This approximation is valid near steady state, where each of the clones is substantially filled. In this approximation, the dynamic equations for, eg, the clone proportions 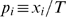 are
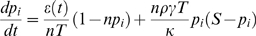
(2)where 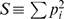 is recognized as Simpson's Index [53].

Using this same approximation, the dynamics of the total T-cell population size is given by
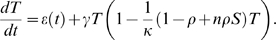
(3)


At the single stable steady state, which is unique and stable, all clones have the same size, independent of 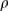. Therefore, the steady-state solution is 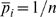 for each *i*, 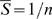, and 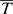 is given by the positive solution of 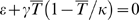 (Text S1).

When 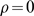, all growth limitation is due to competition for TCR-nonspecific resources while spMHC is plentiful. Under these conditions, the total population growth decouples from the diversity (as measured by the Simpson index). At 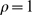, all growth limitation is due to competition for spMHC, while TCR-nonspecific resources are abundant and the effective clones grow independently of one another.

Because the thymic graft is likely to become functional only gradually over time, we model the thymic emigration rate as a Hill function,
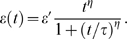
(4)


This parameterization is atypical, but proves to be practical for performing the fitting, as will be described in the Results section. 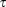 is the time in days at which thymic output is half-maximal, and the Hill coefficient, 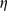, fixes the steepness of the curve at the half-maximum emigration rate.

Sample paths under the model are generated using software written in C# under Visual Studio 2008, and the Troschuetz pseudorandom number generator classes (http://www.codeproject.com/KB/recipes/Random.aspx). The continuous components are integrated numerically using a fourth-order Runge-Kutta scheme with adaptive step size. Thymic emigration is implemented as follows. For each time interval 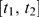, used for the numerical integration, we integrated inhomogeneous Poisson intensity
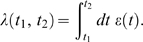
(5)


We then draw a Poisson random variable, call it 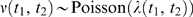. Finally, we add 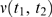 clones of size 1 to the system at time 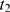. For each new clone, the T-cell receptor beta V (TCRBV) family and CDR3 length conditional on TCRBV are assigned at random using multinomial parameters estimated using data from several healthy subjects.

### Subject Data

#### Ethics statement

This study was conducted according to the principles expressed in the Declaration of Helsinki. All subject samples were obtained under protocols approved by the Duke University Medical Center Institutional Review Board (IRB).

Between 13 and 17 blood samples per subject were collected over a period of 2–3 years following thymus transplantation in three infants with complete DiGeorge anomaly. T cell receptor diversity in CD4 T cells was assessed by obtaining frequencies of TCRBV family usage by flow cytometry, and of complementarity determining region 3 (CDR3) length distributions within TCRBV families by spectratyping and using these frequencies to estimate the diversity as described below.

Standard flow cytometry on whole blood samples was performed as previously described [44]. Monoclonal antibodies against CD3, CD4, and CD8 were used in conjunction with antibodies specific to 18 TCRBV families (Beckman Coulter and BD Biosciences).

**Figure 2:**
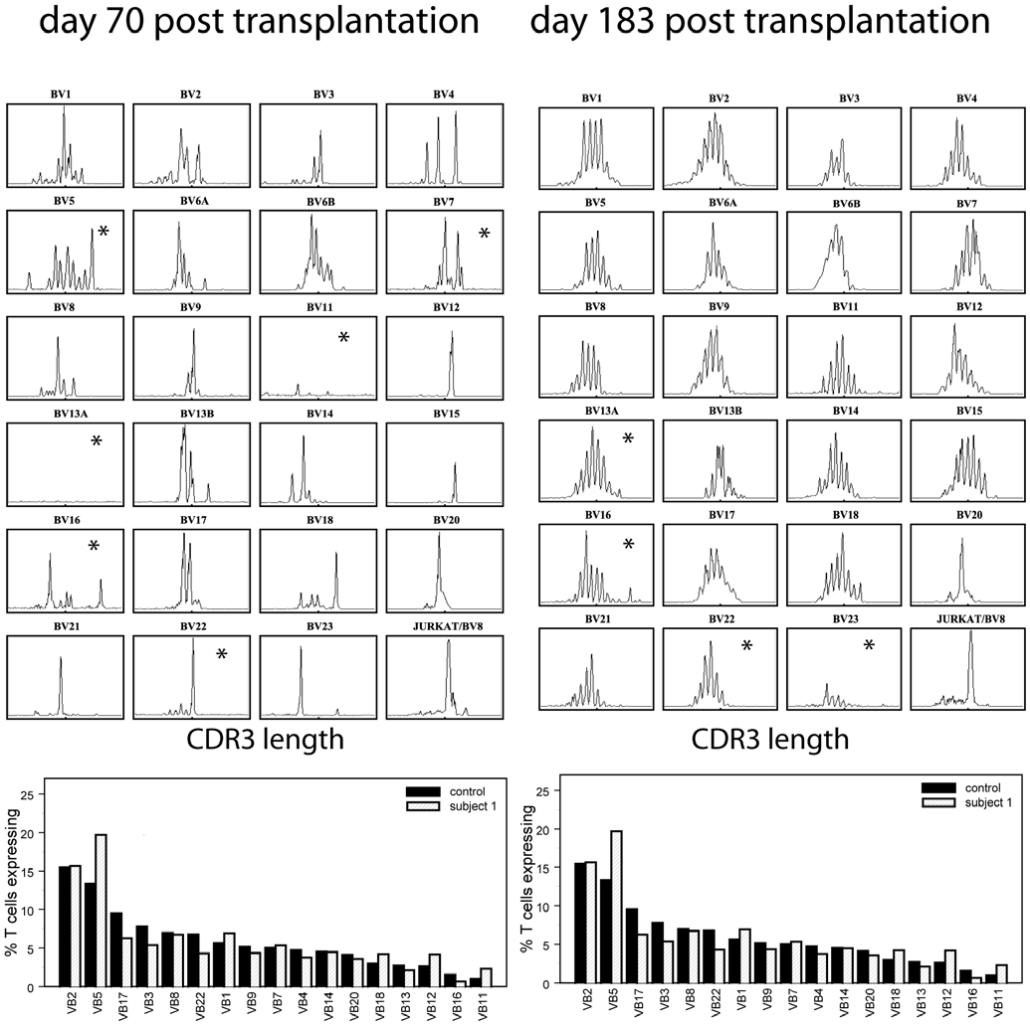
Spectratype data. Raw CD4^+^ spectratype data (upper panel) for subject 1 on days 70 (left) and 183 (right) post-transplantation. CD4^+^ TCRBV usage frequency for average over 10 healthy controls (solid bars), and subject 1 (striped bars) on the same two days. The raw spectratype profiles are not represented on a consistent scale. Assays that had no peaks above 500 fluorescent units are routinely excluded from subsequent analysis. These are marked with an asterisk.

Spectratype analysis provided information about CDR3 length diversity within each functional TCRBV family. Briefly, CD4 T cells were isolated from the peripheral blood of subjects and controls. RNA was prepared and used for complementary DNA (cDNA) synthesis. The cDNA was used as a template for 23 TCRBV-specific primer pairs covering 21 TCRBV families to amplify the complete CDR3 region by PCR [54]. Each PCR product, representing a different TCRBV family, was size separated by capillary gel electrophoresis and the product lengths were identified using the GeneScan software (Applied Biosciences). Product length distributions of a Gaussian-like profile correspond to a polyclonal T cell repertoire. Variations between spectratype histograms of DiGeorge subjects and those of healthy adult controls provide information about the diversification of the TCRBV repertoire over time following transplantation. The raw spectratype densitograms and TCRBV family frequency comparisons taken at two times post-transplantation from one subject are displayed in Figure 2.

### Quantification of Diversity

The Kullback-Leibler divergence (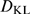) is a measure of the difference between two probability mass functions that arises naturally in the context of likelihood ratio tests for multinomial distributions. The diversity of patient TCR repertoires can be quantified via 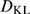-based comparison of patient spectratypes to mean spectratypes averaged over healthy controls [55], and 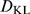-based comparison of TCRBV frequencies to those in healthy controls.

Furthermore, 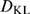 can be decomposed hierarchically into components attributable, respectively, to variation in TCRBV family usage, CDR3 length variation within TCRBV family, and protein sequence variation within CDR3 length. For this work, we measure TCRBV family usage by flow cytometry and distributions of CDR3 lengths by spectratyping. The fact that we are not analyzing sequence data implies that we are not making the highest-resolution diversity measurements possible. We will show, however, that this potential limitation does not impede our ability to perform the measurements we set out to make. The data obtained in each of these assays represents the relative frequency of TCR counts in a subclass conditional on the parent class: 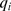 is the fraction of cells using a gene segment from TCRBV family 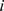 in a given DiGeorge subject and 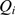 is the mean usage in our collection of healthy controls. 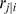, and 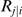 are the relative numbers of T cells of CDR3 lengths 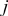 within those cells using TCRBV family 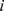 in a given patient, and among controls, respectively.

The 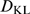 at the level of TCRBV is
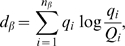
(6)where 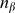 is the number of TCRBV families considered, which in our case is 23. The CDR3-length divergence within the 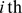 family is
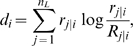
(7)where 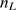 is the number of CDR3 lengths, in our case 18. The total divergence is
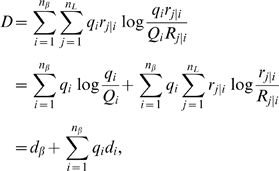
(8)


The inverse of the 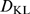 can be regarded as the “completeness” of a TCR repertoire with respect to some standard [55]. Under very reasonable assumptions, the completeness may be regarded as a measure of the diversity, so we will refer to the diversity of the TCR repertoire, and quantify it by the sample completeness, 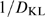. Note that diversity increases as the the 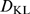 decreases toward its minimum value, zero.

The sample 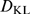, like the sample entropy, is a biased estimator of the population 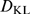: the expected value of the sample 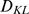 differs from the population 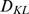 by an additive term inversely proportional to the sample size. We account for this fact by including an additive bias as a parameter 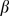 in the estimation procedure. We can then compare the estimated bias against the sample sizes (Table 1) for each datum.

**Table 1:** Sample sizes for the biological assays used to collect data on the three DiGeorge subjects.

Subject	days post-transplantation	# cells/RT reaction	CD4^+^ T cell events
1	70	63,636	3,766
	88	200,000	1,130
	117	38,889	4,437
	145	125,000	6,396
	183	58,696	8,106
	398	441,176	17,261
2	175	109,091	20,302
	209	71,429	26,329
	286	67,273	36,712
3	102	126,667	9,090
	130	230,000	15,058
	166	51,020	20,669
	372	892,857	43,371

“# cells/RT reaction” is the number of cells used in RNA extraction for spectratyping. The numbers were estimated from the starting number of CD3^+^ cells and RNA yield. All spectratyping used 150 ng of RNA regardless of the cellular sample size. “CD4^+^ T cell events” refers to the total number of flow cytometric events in the CD3^+^CD4^+^ gate used to determine the proportion of cells in each of 22 TCRBV families as described in the text.

### Parameter Fitting

The model is based on absolute T-cell numbers, but the data are blood T cell concentrations. We must use a conversion factor 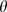 that converts total T cell numbers to T-cell blood concentration. This factor is not easy to estimate with any precision, but our results are quite insensitive to large variation in 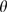.

Assuming that a 10 kg subject has 600 ml of blood, and that there are 45 times more lymphocytes in the tissues than in the blood, based on adult data [56], we have 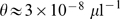. This is the conversion factor we use in the rest of the paper.

Furthermore, some subjects have a relatively small number of anomalous T cells at the time of transplantation. We denote the concentration of such cells 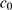 and treat them as a separate non-functional family. The model quantity ultimately used for comparison to patient T-cell concentration data is 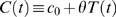.

We estimate the remaining parameters 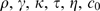, and 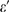, as well as the measurement error variances 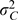 and 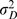, and the 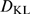 measurement bias *β*, by fitting the model specified by Eq.(1) to the patient T cell concentration and TCR diversity data simultaneously using Markov Chain Monte Carlo (MCMC; see, eg, [57]).

We estimate parameters by using the likelihood function given by
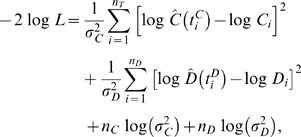
(9)where 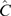 and 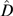 are the estimated T cell concentrations and diversities, respectively, under the model specified by Eqs.(1,4); 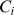 and 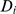 are the corresponding patient data observed at sampling times 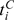 and 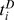, respectively. 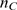 and 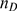 are the total number of T cell concentration and diversity data points, respectively; 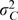 and 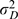 are the error variances. We compute the posterior joint density of all model parameters using a block-sampled Metropolis-Hastings MCMC.

The parameters are all manifestly positive, and 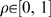. Therefore, we transformed 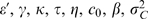, and 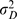 using natural logs. 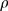 was treated using a logistic transformation. 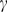 represents the maximum growth rate of T cell populations. The maximum possible rate is set by the minimum cell-cycle time for lymphocytes of about 6 hours, so we likewise used a logistic transformation on 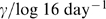. The prior distributions on the parameters were uniform in the transformed variables.

## Results

### Parameter Estimates

The maximum-likelihood parameters estimates are shown in Table 2, along with the 95% credible intervals. The estimate for 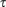 was much larger than the largest observation time, so we subsequently fixed 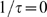, thereby eliminating one modeling degree of freedom and refit the model. We found that this simplification entailed no significant decrease in likelihood for any data set. This result implies that our experimental design is not capable of resolving the full time-course of the grafts' becoming functional, not necessarily that the emigration rate continues to increase indefinitely.

**Table 2:** Best fit estimates.

	meaning	units	subject		
			1	2	3
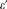	thymic emig. amplitude	10^−4^ cells/day*^η^*	6.5, (6.2,6.9)	9.5, (8.4,10)	21.5, (16.9,21.8)
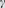	max. growth rate	day^−1^	2.67, (2.58,2.72)	0.52, (0.41,0.72)	0.45, (0.39,0.98)
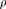	carrying capacity ratio	10^−4^	6.2, (5.2,7.7)	27, (25,58)	1.4, (1.4,3.4)
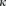	carrying capacity	10^10^	2.6, (2.4,2.7)	3.7, (3.3,3.9)	2.9, (2.4,3.3)
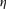	emigration rate exponent	1	1.9, (1.8,2)	1.9, (1.9,2.1)	1.9, (1.6,2.1)
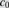	baseline T cell conc	cells/µl	59, (53,60)	10, (10,11)	26, (21,35)
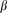	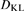 bias	1	0.07, (0.069,0.081)	0.077, (0.074,0.1)	0.12, (0.11,0.23)
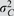	error variance, T cell conc	(cells/µl)^2^	13, (13,16)	1.8, (1.7,2.4)	16, (16,34)
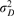	error variance, 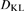	10^2^	10, (10,16)	0.38, (0.07,0.58)	19, (19,56)

Maximum-likelihood estimates and upper (UL) and lower (LL) limits on the 95% credible intervals for model parameters.

The estimate of greatest interest for us is 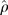, which we find lies between about 10^−4^ and 10^−3^ in these three subjects. In subject one, who has the most complete data available and the tightest posterior marginal distribution on 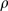, produces an estimate midway between the other two.

The parameter governing total thymic emigration, 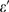, does not have a simple direct interpretation, but one can estimate the thymic emigration rate at any time post-transplantation. These rates, estimated for the three subjects at one year post-transplantation, are 93, 44, and 198 cells per day, respectively. These rates are several orders of magnitude smaller than those estimated for healthy humans infants.

We can examine the estimated values of the the 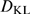 bias and use the known expression for this bias to compare to the actual sample sizes. Where the underlying distribution is multinomial, the theoretical bias is given by 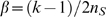 where 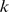 is the number of classes in the multinomial, and 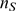 is the sample size. Using this formula to estimate the sample size leads to the conclusion that the effective sizes of the samples used to estimate diversity are between 10^3^ and 10^5^ cells, consistent with the measured sample sizes (Table 1).

**Figure 3:**
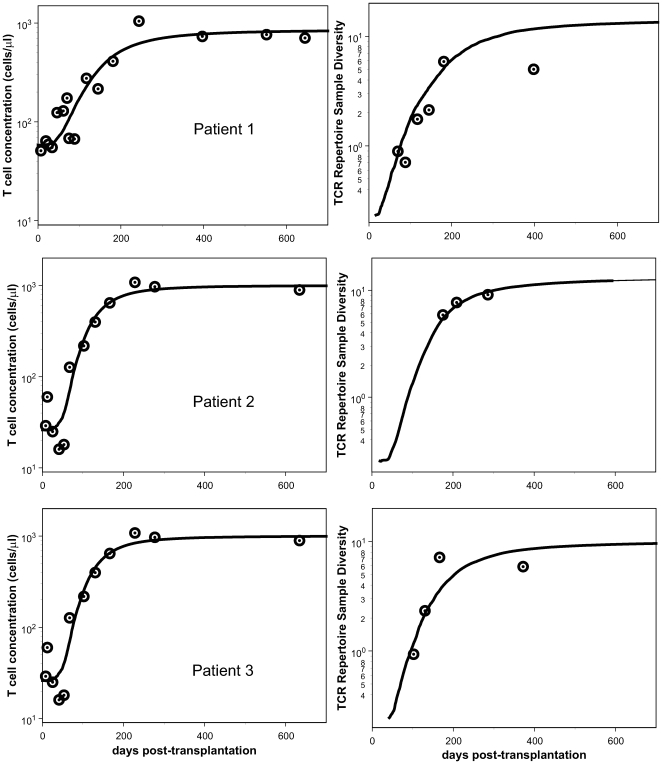
Best fit to the data. Maximum-likelihood fits of the population-dynamic model given by Eqs.(1,4) to patient data. The curves are the trajectories of 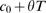 and 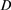, respectively.

Figure 3 provides a visual assessment of the quality of the model fits to these data.

In order to gain a deeper understanding of the role of 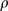 on the dynamics of both T cell population size and diversity, we performed two additional analyses. We use the data from subject one to illustrate these points. Analyses on the other two subjects yielded comparable results and lead to the same conclusions.

**Figure 4:**
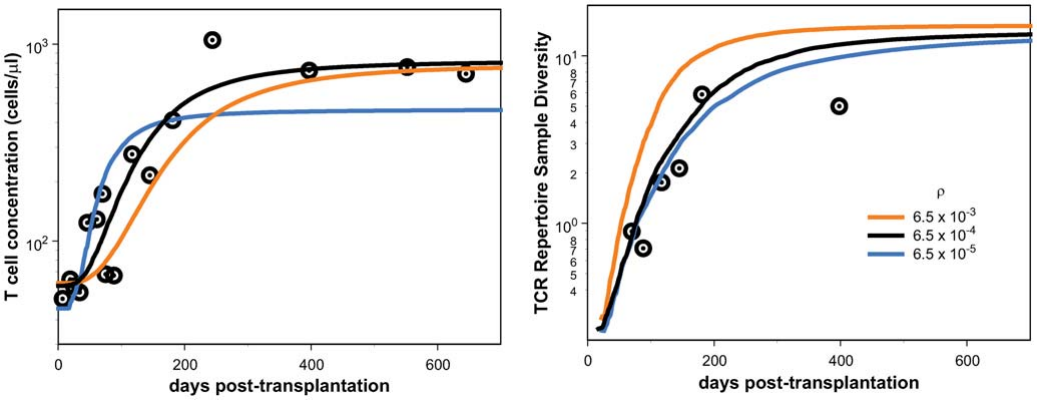
Best fit for constant *ρ*. Maximum-likelihood fit of data from subject 1 to the population-dynamic model given by Eqs.(1,4). The black curve represents the maximum likelihood solution; the orange and blue curves represent the maximum likelihood solutions subject to a constraint on 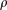 at the values indicated. The curves are the trajectories of 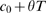 and 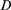, respectively.

A reasonable concern is that thymic emigration rate and 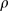 may be confounded–that it may be possible to compensate for smaller 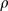 by making 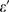 larger. We addressed that concern by performing a numerical experiment to determine the impact on the model fit of fixing 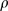 outside the inferred credible bounds. We fix 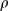 to be 10-fold smaller or larger than its maximum-likelihood estimate for subject 1. The fits are significantly worse (compare Figures 3 and 4), with log-likelihood ratios of about 8 in both cases (Table 3).

**Table 3:** Maximum-likelihood estimates in the constrained model with fixed *ρ*.

Model	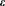	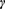	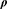	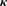	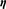	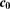	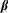	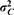	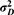	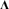
	10^−4^ cells/day*^η^*	day^−1^	10^−4^	10^10^ cells		cells/µl		(cells/µl)^2^		
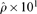	10	2.6	65	2.6	2.3	51	0.073	0.15	1.27	8.7
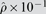	5.9	2.5	0.65	1.4	1.9	46	0.069	0.32	0.12	7.7

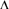
 is the log of the likelihood ratio.

### Sensitivity Analysis

A focused analysis of the time-dependent sensitivity of the model trajectories to parameter variation may provide further insight into the mechanisms of regulation. We examined the sensitivity of our model to changes in 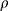 [58],[59] and 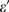 as follows.

Define the sensitivity functions 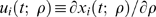, and 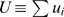. The sensitivity equations are obtained by differentiating both sides of equation (1) with respect to 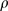. The 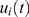 are computed as the solutions of
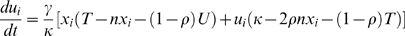
(10)with 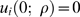 for all *i*. The sensitivity function for *D* is given by 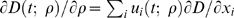. The relative sensitivities are calculated by multiplying the absolute sensitivity by 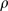 and dividing by the value of the relevant response variable.

The sensitivity to 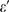 is due largely to the changed timing of discrete events, and so does not lend itself easily to differential sensitivity analysis in the form outlined in Eq(10). Sensitivity to 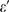 was therefore estimated directly by generating sample paths under different values of 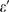.

**Figure 5:**
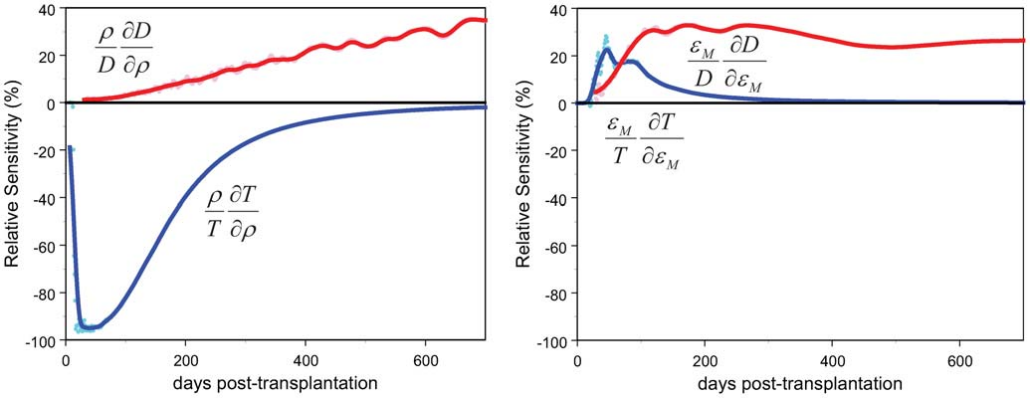
Sensitivity curves. Relative sensitivity trajectories for the model fit to data from subject 1. The left panel shows the relative sensitivity to changes in *ρ*; the right panel shows the relative sensitivity to changes in 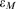, the maximum thymic emigration rate. Blue curves give the model solutions for *c*_0_+*θT*; red curves give the model solutions for the (unbiased) TCR repertoire diversity.

We know that mean sensitivity to 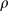 is transient because the steady-state population size and diversity do not depend on 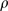 in the deterministic limit (Eqs.2,3). We find, however, that the diversity remains sensitive to changes in 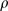 throughout the two-year period of the study (Figure 5). It should be noted, however, that this assessment is based on the true diversity rather than the sample diversity, whose resolution is limited by sample size. At the sample sizes available, the sensitivity of the measurable diversity to 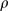 vanishes between 6 and 12 months. The population size is very sensitive to 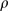 in the first 3–4 months, becoming less so rapidly after that time. As 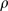 increases, the population size decreases during the sensitive period.

Increases in the thymic emigration rate 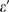 have a positive effect on both the population size and the diversity, as expected. In this case, the sensitivity of the diversity is greatest earlier than in the case of *ρ*. It should be noted that diversity at the level of amino acid sequence differences is not resolved by our assays, regardless of sample size. If it were, we would expect the diversity to remain sensitive to both of these parameters for a longer period of time.

### Response to Clonal Perturbation

The model does not account for the micro-environmental perturbations invariably encountered by people, including our study subjects. Infection produces a transient change in the steady state of the T cell repertoire, typified by a decrease in the diversity. Once the infection has been resolved, the T-cell population returns to its steady state, and the diversity relaxes back to its original value. The rate at which the return to steady state values occurs depends on *ρ*. To examine this dependence, we carried out the following numerical experiment.

**Figure 6:**
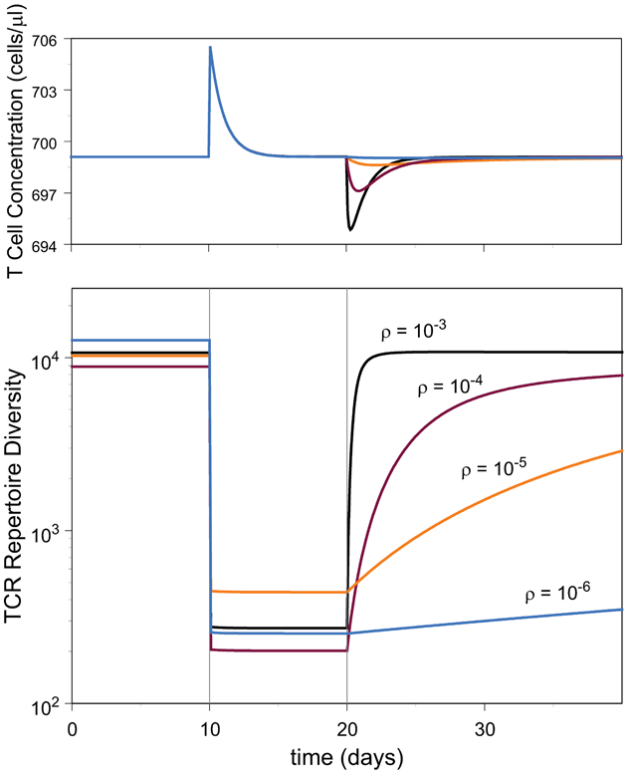
Response of the system to clonal perturbation. The system was initialized at steady state, and was subjected to the sudden enlargement of a single randomly chosen clone by 10,000-fold. This clone was held artificially high for 10 days, after which the system was allowed to relax back to steady-state. The artificially enlarged clone consumes TCR-non-specific resources at a rate appropriate to its size. The TCR repertoire diversity is shown in the bottom panel, the T cell concentration is shown in the panel above. 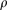 varies as indicated. The variability un the steady state diversity is due to the inherent variability in the system rather than to the imposed changes in 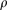.

We start with a system at steady state, and suddenly increase the size of a single clone by a factor of 10^4^, allowing it to consume the non-TCR specific resources its new size requires. The diversity decreases as a result. Over a few days, the other clones adjust to the new steady state. After ten days we remove the artificial support for the enlarged clone and allow the system to return to steady state (Figure 6). We ran this experiment using models with four values of *ρ* differing over four orders of magnitude. For high *ρ*, corresponding to a high competition for specific signals, the diversity decreases less than in the absence of intra-clonal competition. Moreover, after the perturbation is resolved, the diversity increases back to its steady-state, pre-perturbation value at a rate that depends very sensitively on 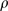. The return to steady-state diversity is more rapid the greater the competition for TCR-specific signals. The estimated values for 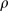 obtained from the DiGeorge patients implies a diversity-return time on the order of a few days, rather than weeks or more.

## Discussion

The functional integration of the manifold processes involved in the development and maintenance of the mature T cell repertoire has not yet been fully elucidated. These processes include thymic export, competition for TCR ligands, and competition for non-specific stimulatory factors. Here, we study T cell homeostasis in complete DiGeorge Anomaly patients during the establishment of T cells following thymic transplantation.

To quantify the balance between TCR-specific and TCR-nonspecific factors that act to limit T-cell population growth, we developed a mathematical model that accounts for intra- and inter-clonal competition. The key parameter in this model for the present purpose is *ρ*. This parameter can be given a functional interpretation by considering the case of an individual capable of rearranging just a single TCR. The size of this hypothetical sole clone, at equilibrium, must be larger than the size of the same clone in the presence of a T cell population with a complete repertoire. The ratio of these two sizes is 1∶*ρ*. We have estimated 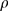 to be about 10^−3^; the carrying capacity for a single isolated clone is about 1000 times larger that that for the same clone under normal heterogeneous conditions. This result suggests that TCR-specific resource limitation is relatively unimportant, but this is true only for the total T-cell population size near steady state; away from steady state, TCR-specific regulation may is crucial to the establishment and maintenance of repertoire diversity.

At steady-state, TCR diversity is maintained through competition for self peptide MHC [60], and population numbers are maintained through competition for cytokines and other resources not specific to the TCR [5],[19].

Our models fit the data adequately only when the thymic emigration is an accelerating function of time through the initial post-transplantation phase, as would be expected from the biology of thymus transplantation [61],[62]. The slices of cultured thymus tissue used for transplantation are cultured from 2–3 weeks prior to transplantation. There is dramatic depletion of thymocytes from the tissue, thymic epithelium becomes condensed, and the cortico-medullary distinction is lost. In the first months after transplantation, the epithelium differentiates so that cortex and medulla again are distinct and the epithelium develops its characteristic lacy appearance. During this process, the numbers of thymocytes in the transplanted tissue increases dramatically, from small numbers of scattered thymocytes to densely packed thymocytes throughout the allograft. Thus, the biology predicts an increase in emigration with time after transplantation as normal thymic architecture is reestablished.

The current study was not able to treat naive and memory T cells separately, but doing so is clearly of great interest given the indications that these pools are regulated independently of each other [11]. Furthermore, we were not able to measure thymic emigration directly, though these measurements would be of significant interest given the surprisingly small rates inferred here, and the impact that the reconciliation of this implied inconsistency would have on our understanding of the treatment of athymia and thymic dysfunction. Finally, our model contains terms for the density-dependent competition among T cells (Eq.1) whose forms are commonly accepted in population modeling but do not have direct empirical justification in the present context. Although we believe that our conclusions are robust against reasonable variation in these terms, it would be of great utility to explore this issue explicitly.

Our aim has been to study T cell homeostasis in a more natural human system than has otherwise been available thus far. It remains to be shown just how natural the post-transplantation environment is in this regard, and therefore how broadly applicable our findings are. We are encouraged, however, by the fact that complete DiGeorge patients receiving thymus transplants recover adaptive immune function. It is important to note that total T-cell counts in these recovered patients remains below normal, typically at about the 10th percentile for children of the same age [45]. Although specific reasons for this condition have not yet been determined, no molecular defects in T-cell function have been reported. We regard it as possible that the sole immunological lesion in DiGeorge anomaly is athymia, and that, once ameliorated by transplantation, the emergence of the T cell population proceeds as it would in a non-affected T-cell lymphopenic subject. We expect, therefore, that our findings will be generally applicable.

Our study enabled us to discriminate between two qualitatively different forms of population regulation: TCR-specific regulation and TCR-nonspecific regulation, and to elucidate their roles in jointly maintaining the dynamic homeostasis of the T cell population. The parameters estimates obtained by analyzing data from three DiGeorge Anomaly patients suggest that T-cell population size is maintained by TCR-nonspecific mechanisms, and TCR repertoire diversity is maintained by TCR-specific mechanisms.

## Supporting Information

Text S1 Supporting Information
(0.06 MB PDF)
