# Supplementary material for: The Dynamics of T-Cell Receptor Repertoire Diversity Following Thymus Transplantation for DiGeorge Anomaly
Source: PLoS Comput Biol. 2009 Jun 12;5(6):e1000396. doi: 10.1371/journal.pcbi.1000396 (PMC2690399; doi:10.1371/journal.pcbi.1000396)
Supplement: Text S1 — Supporting Information (0.06 MB PDF) [file pcbi.1000396.s001.pdf]

The dynamics of T-cell receptor repertoire diversity following thymus transplantation for DiGeorge Anomaly- Text S1

Stanca M. Ciupe*, **Blythe H. Devlin**[†], **M. Louise Markert**[†,‡] , **Thomas B. Kepler***[,‡]

Because the number of lineages is large and because we study the long-term average behavior of x_i we can replace the stochastic terms by their expectation per unit time to study the average or steady-state behavior. At steady-state, all clones have the same size. Indeed, if we consider that thymic emigration has attained its maximal value $\epsilon_M = \epsilon/\tau^\eta$, we can derive the steady state solution for a family x_i to be

$$\overline{x}_i = \frac{\gamma - \frac{\gamma(1-\rho)}{\kappa}\overline{T} + \sqrt{(\gamma - \frac{\gamma(1-\rho)}{\kappa}\overline{T})^2 + 4\epsilon_M \frac{n\gamma\rho}{\kappa}}}{2\frac{n\gamma\rho}{\kappa}}, \tag{1}$$

which is independent of specificities i. Therefore at equilibrium all clone sizes are identical and equal to $\overline{x}_i = \overline{T}/n$. The total number of T cells satisfies the equation

$$n\epsilon_M + \gamma\overline{T} - \frac{\gamma}{\kappa}\overline{T}^2 = 0. \tag{2}$$

Moreover, if the thymic contribution to the peripheral pool is negligible at steady state, $\epsilon_M \ll \gamma\overline{T}$, the total number of T cells at equilibrium is

$$\overline{T} = \kappa. \tag{3}$$

We can show that the steady state $(\overline{T}/n, \overline{T}/n, ..., \overline{T}/n)$ is asymptotically stable. We linearize Eqs.(1,4) about the steady state (1) to get

$$A = \begin{pmatrix} \gamma - \gamma\frac{(2n-1)\rho+1}{\kappa}\overline{x}_1 - \frac{\gamma(1-\rho)}{\kappa}\overline{T} & -\frac{\gamma(1-\rho)}{\kappa}\overline{x}_2 & ... & -\frac{\gamma(1-\rho)}{\kappa}\overline{x}_n \\ -\frac{\gamma(1-\rho)}{\kappa}\overline{x}_1 & \gamma - \gamma\frac{(2n-1)\rho+1}{\kappa}\overline{x}_2 - \frac{\gamma(1-\rho)}{\kappa}\overline{T} & ... & -\frac{\gamma(1-\rho)}{\kappa}\overline{x}_n \\ ... & ... & ... & ... \\ -\frac{\gamma(1-\rho)}{\kappa}\overline{x}_1 & -\frac{\gamma(1-\rho)}{\kappa}\overline{x}_2 & ... & \gamma - \gamma\frac{(2n-1)\rho+1}{\kappa}\overline{x}_n - \frac{\gamma(1-\rho)}{\kappa}\overline{T} \end{pmatrix}$$
$$= \begin{pmatrix} \gamma - \gamma\frac{(2n-1)\rho+1}{\kappa}\frac{\overline{T}}{n} - \frac{\gamma(1-\rho)}{\kappa}\overline{T} & -\frac{\gamma(1-\rho)}{\kappa}\frac{\overline{T}}{n} & ... & -\frac{\gamma(1-\rho)}{\kappa}\frac{\overline{T}}{n} \\ -\frac{\gamma(1-\rho)}{\kappa}\frac{\overline{T}}{n} & \gamma - \gamma\frac{(2n-1)\rho+1}{\kappa}\frac{\overline{T}}{n} - \frac{\gamma(1-\rho)}{\kappa}\overline{T} & ... & -\frac{\gamma(1-\rho)}{\kappa}\frac{\overline{T}}{n} \\ ... & ... & ... & ... \\ -\frac{\gamma(1-\rho)}{\kappa}\frac{\overline{T}}{n} & -\frac{\gamma(1-\rho)}{\kappa}\frac{\overline{T}}{n} & ... & \gamma - \gamma\frac{(2n-1)\rho+1}{\kappa}\frac{\overline{T}}{n} - \frac{\gamma(1-\rho)}{\kappa}\overline{T} \end{pmatrix}. \tag{4}$$

The characteristic equation for the linearized system is

$$(\gamma - \frac{2\gamma}{\kappa}\overline{T} - \Lambda)(\gamma - \frac{\gamma(1+\rho)}{\kappa}\overline{T} - \Lambda)^{n-1} = 0. \tag{5}$$

From equation (2) we have that $\gamma - \frac{\gamma}{\kappa}\overline{T} = -n\epsilon_M/\overline{T}$. We can conclude that all eigenvalues of (5),

$$\begin{aligned} \Lambda_1 &= -\frac{\gamma}{\kappa}\overline{T} - \frac{n\epsilon_M}{\overline{T}}, \\ \Lambda_{2,3...n} &= -\frac{\gamma\rho}{\kappa}\overline{T} - \frac{n\epsilon_M}{\overline{T}}, \end{aligned} \tag{6}$$

are negative, therefore the steady state $(\overline{T}/n, \overline{T}/n, ..., \overline{T}/n)$ is asymptotically stable.
